# Supplementary figures and images for: A Biological Signature for the Inhibition of Outer Membrane Lipoprotein Biogenesis
Source: mBio. 2022 Jun 13;13(3):e00757-22. doi: 10.1128/mbio.00757-22 (PMC9239194; doi:10.1128/mbio.00757-22)

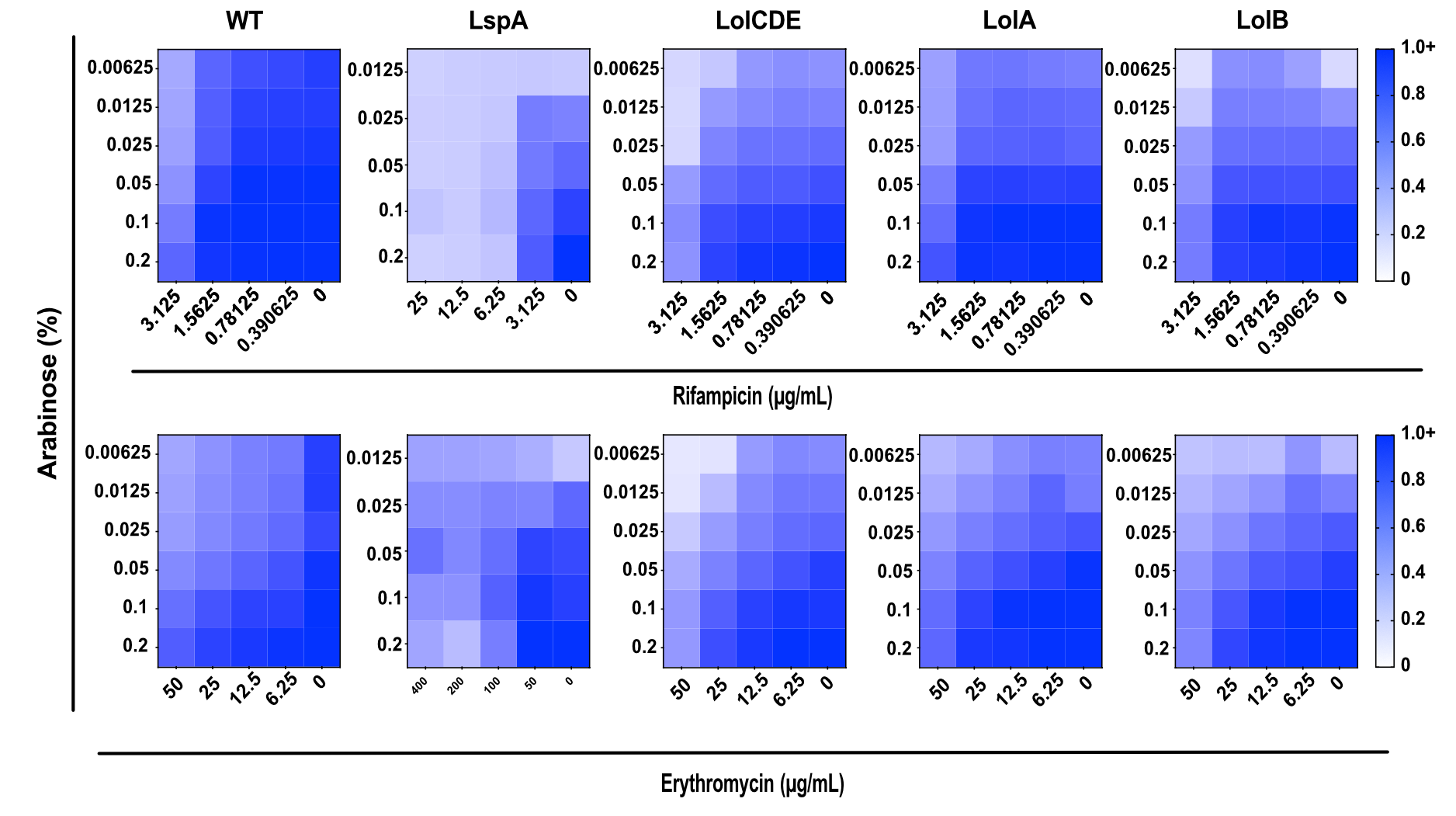

Supplement: FIG S1 [file mbio.00757-22-s0001.tif]

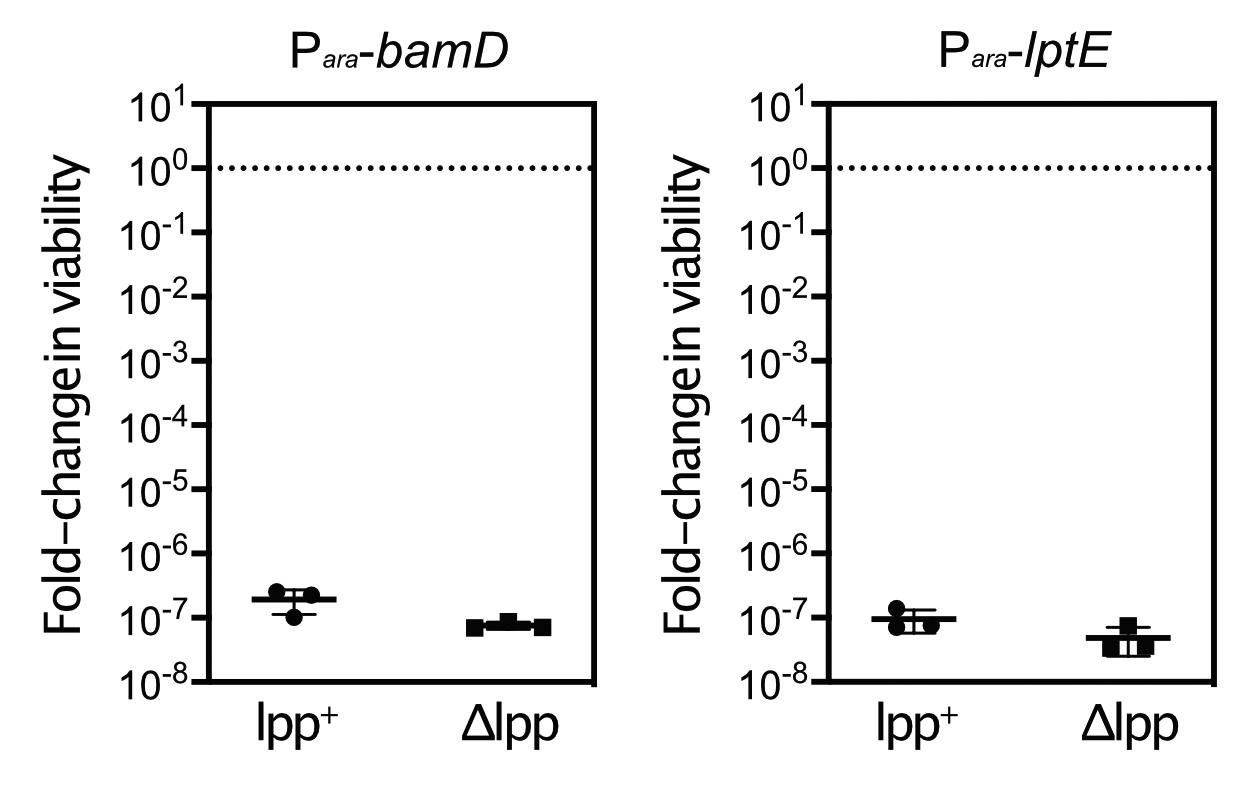

Supplement: FIG S2 [file mbio.00757-22-s0002.tif]

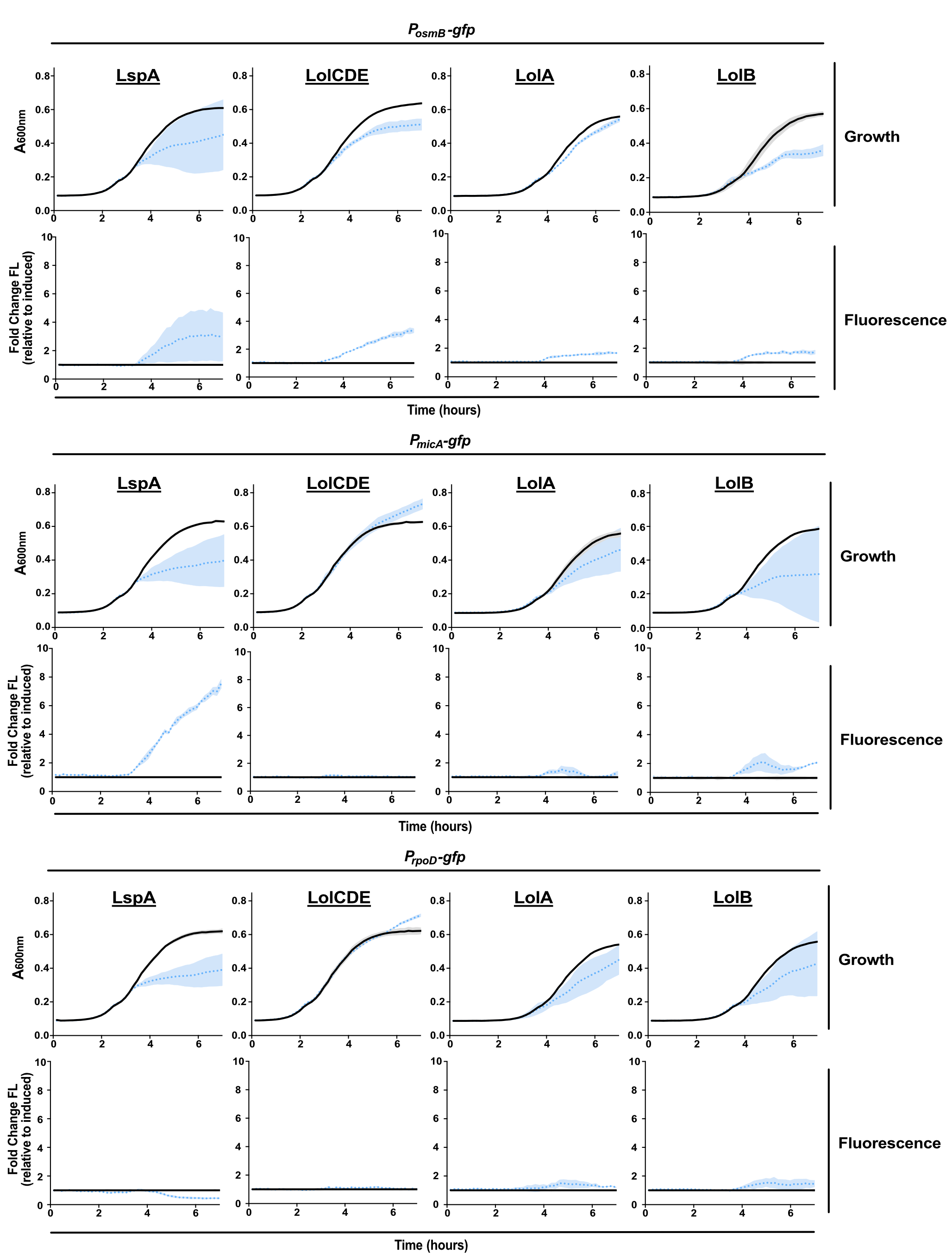

Supplement: FIG S3 [file mbio.00757-22-s0003.tif]

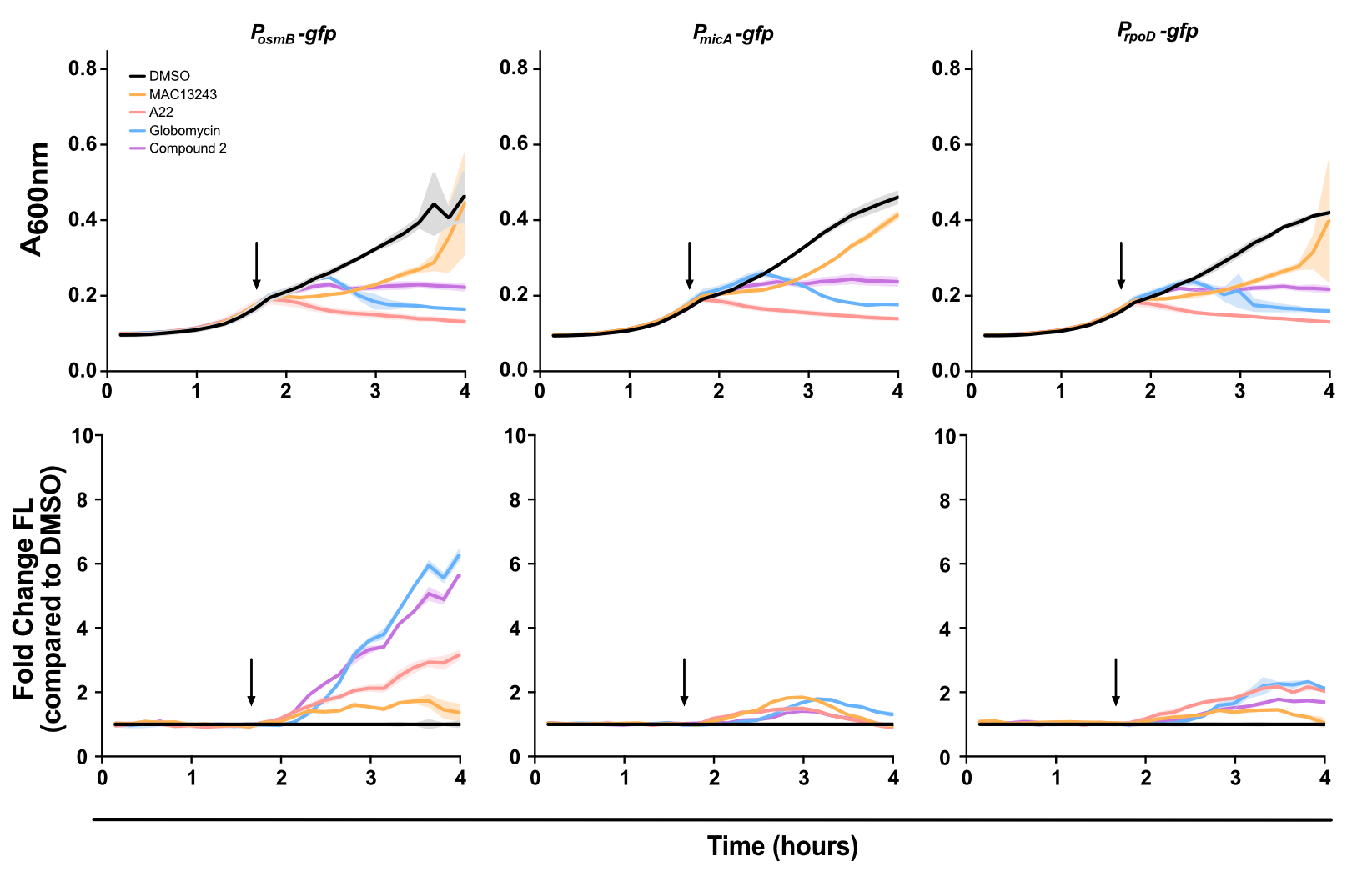

Supplement: FIG S4 [file mbio.00757-22-s0004.tif]

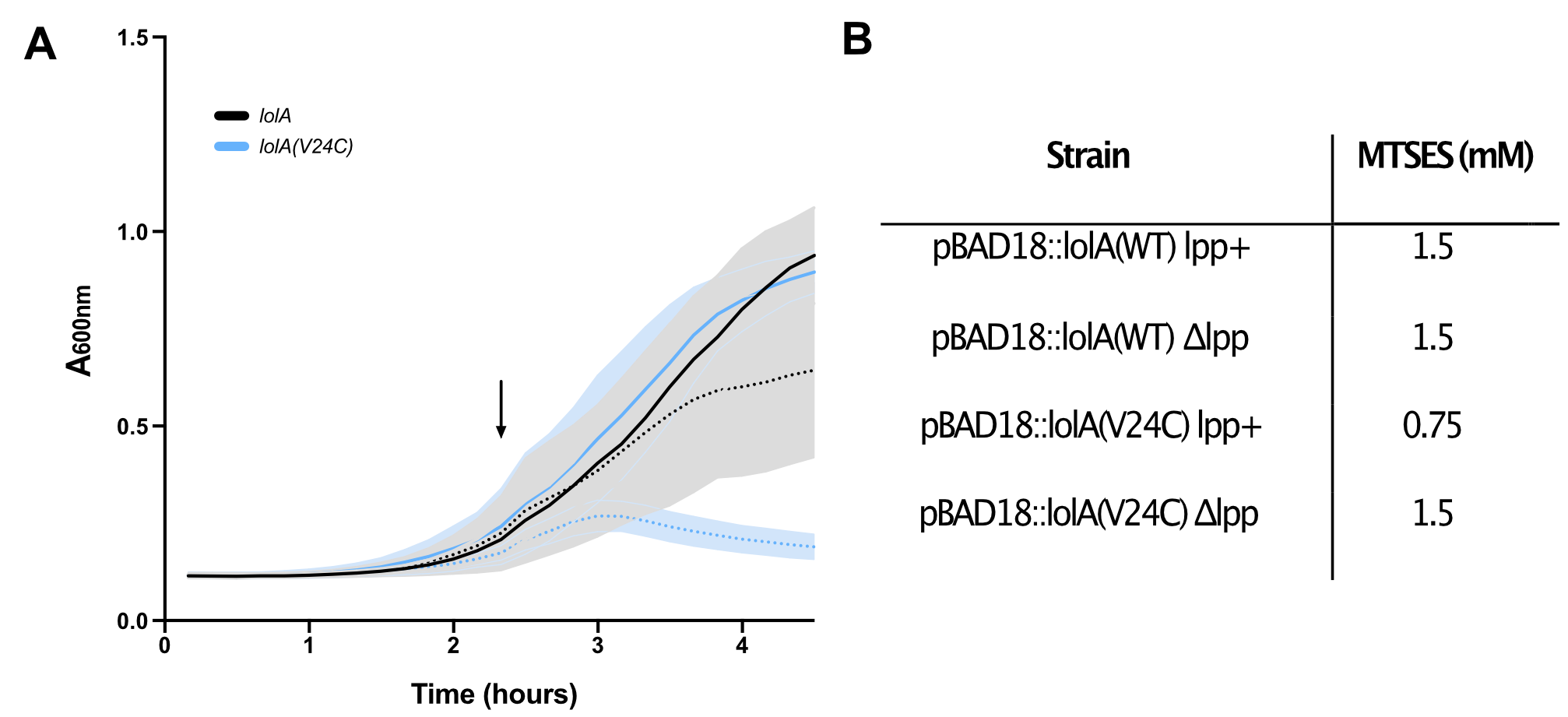

Supplement: FIG S5 [file mbio.00757-22-s0005.tif]

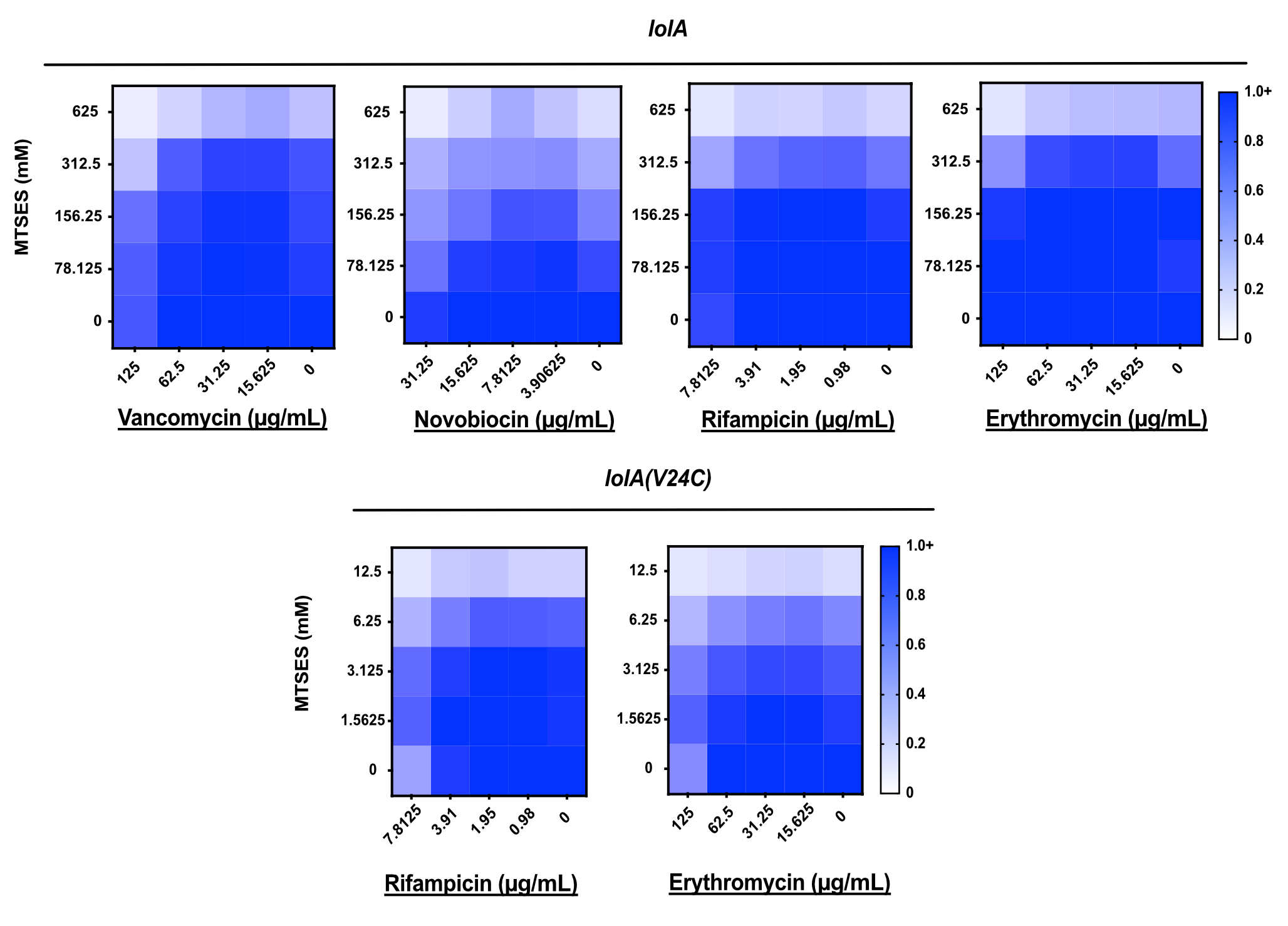

Supplement: FIG S6 [file mbio.00757-22-s0006.tif]

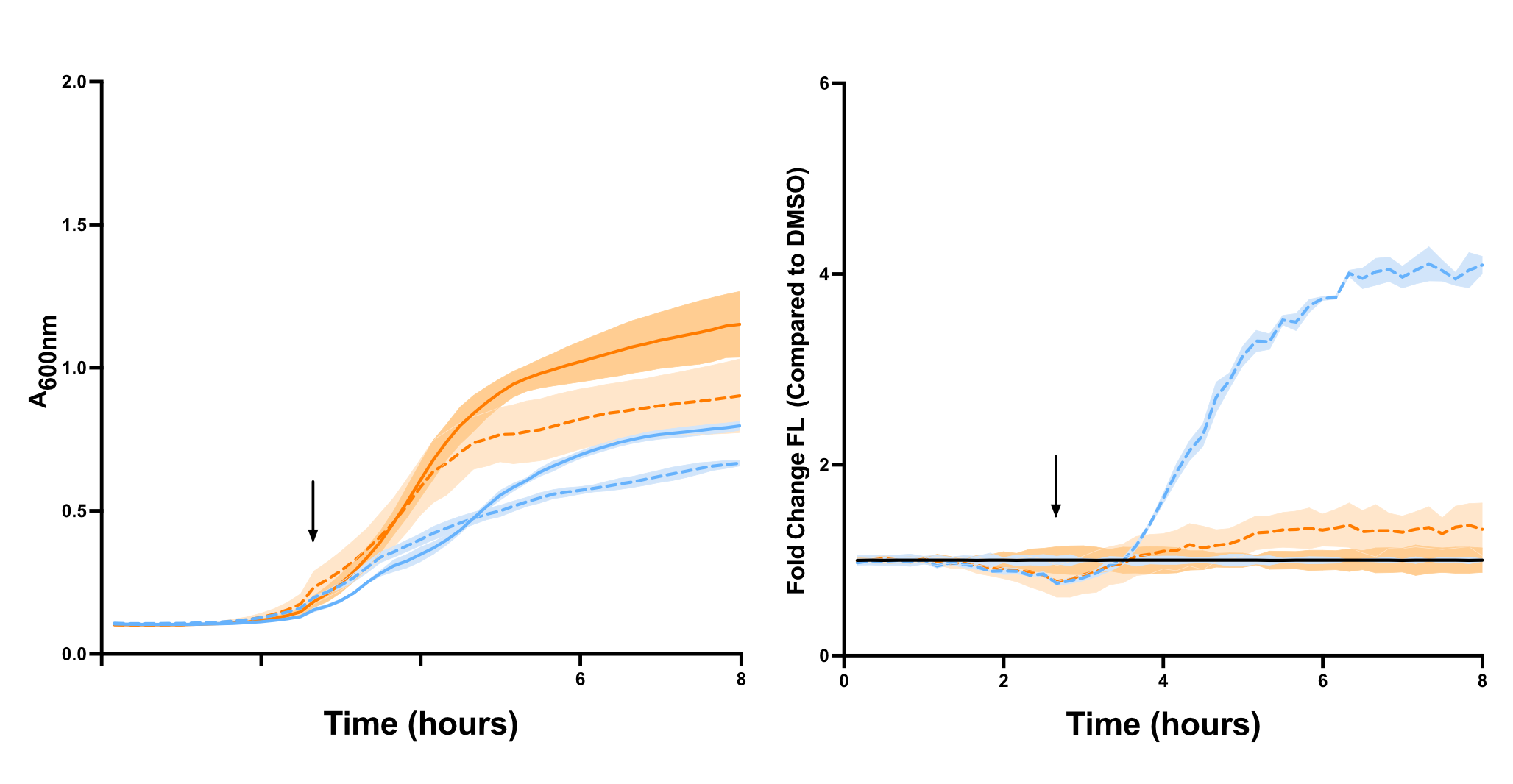

Supplement: FIG S7 [file mbio.00757-22-s0007.tif]

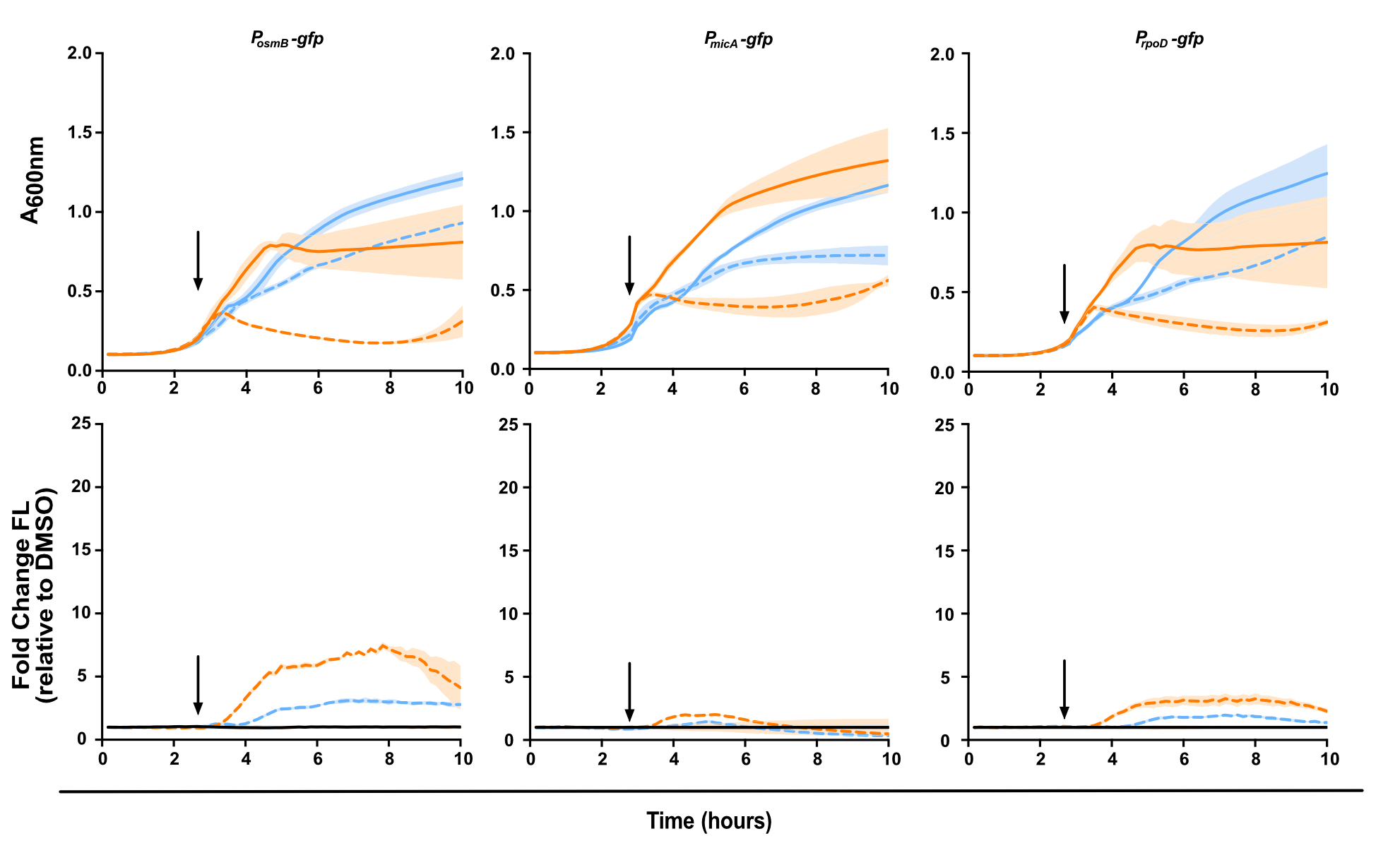

Supplement: FIG S8 [file mbio.00757-22-s0008.tif]
