# Supplementary material for: A Biological Signature for the Inhibition of Outer Membrane Lipoprotein Biogenesis
Source: mBio. 2022 Jun 13;13(3):e00757-22. doi: 10.1128/mbio.00757-22 (PMC9239194; doi:10.1128/mbio.00757-22)
Supplement: TABLE S1 [file mbio.00757-22-s0009.docx]

**Table S1: Strains List**

| Strain Name | Genotype | Use/Description | Reference |
| --- | --- | --- | --- |
| MC4100 | MC4100 F- {[*araD*139]B/r Δ(*argF*-*lac*)169 λ- e14- *flhD*5301 Δ(*fruK*-*yeiR*)725(*fruA*25) *relA*1 *rpsL*150(*strR*) rbsR22 thi-1 Δ(*fimB*-*fimE*)632(::IS1) *deoC*1} | WT *E. coli* K-12 | 1 |
| NR756 | MC4100 ara^+^ | MC4100 isolate able to catabolize arabinose | 2 |
| MG3178 | MC4100 ara^R^ | MC4100 isolate able to grow in the presence arabinose | 3 |
| Gene Depletion and Stress GFP Reporter Strains | | | |
| KL864 | NR756 ∆*lspA* [pBAD30::*lspA*] [pUA66::P*_cpxP_*-GFP] | LspA depletion, stress response reporter assay | This Study |
| KL865 | NR756 ∆*lspA* [pBAD30::*lspA*] [pUA66::P*_osmB_*-GFP] | LspA depletion, stress response reporter assay | This Study |
| KL866 | NR756 ∆*lspA* [pBAD30::*lspA*] [pUA66::P*_micA_*-GFP] | LspA depletion, stress response reporter assay | This Study |
| KL867 | NR756 ∆*lspA* [pBAD30::*lspA*] [pUA66::P*_rpoD_*-GFP] | LspA depletion, stress response reporter assay | This Study |
| KL984 | NR756 ∆*lspA* *nlpE*::*spec* [pBAD30::*lspA*] [pUA66::P*_cpxP_*-GFP] | LspA depletion, NlpE deletion, stress response reporter assay | This Study |
| KL822 | NR756 Δ*lolCDE::cam* [pBAD18::*lolCDE*] [pUA66::P*_cpxP_*-GFP] | LolCDE depletion, stress response reporter assay | This Study |
| KL823 | NR756 Δ*lolCDE::cam* [pBAD18::lolCDE] [pUA66::P*_osmB_*-GFP] | LolCDE depletion, stress response reporter assay | This Study |
| KL824 | NR756 Δ*lolCDE::cam* [pBAD18::*lolCDE*] [pUA66::P*_micA_*-GFP] | LolCDE depletion, stress response reporter assay | This Study |
| KL825 | NR756 Δ*lolCDE::cam* [pBAD18::*lolCDE*] [pUA66::P*_rpoD_*-GFP] | LolCDE depletion, stress response reporter assay | This Study |
| KL982 | NR756 Δ*lolCDE::cam* *nlpE::spec* [pBAD18::*lolCDE*] [pUA66::P*_cpxP_*-GFP] | LolCDE depletion, NlpE deletion, stress response reporter assay | This Study |
| KL860 | NR756 ∆*lolA* [pBAD30::*lolA*] [pUA66::P*_cpxP_*-GFP] | LolA depletion, stress response reporter assay | This Study |
| KL861 | NR756 ∆*lolA* [pBAD30::*lolA*] [pUA66::P*_osmB_*-GFP] | LolA depletion, stress response reporter assay | This Study |
| KL862 | NR756 ∆*lolA* [pBAD30::*lolA*] [pUA66::P*_micA_*-GFP] | LolA depletion, stress response reporter assay | This Study |
| KL863 | NR756 ∆*lolA* [pBAD30::*lolA*] [pUA66::P*_rpoD_*-GFP] | LolA depletion, stress response reporter assay | This Study |
| KL983 | NR756 ∆*lolA* *nlpE::spec* [pBAD30::*lolA*] [pUA66::P*_cpxP_*-GFP] | LolA depletion, NlpE deletion, stress response reporter assay | This Study |
| KL818 | NR756 ∆(λ*att-lom*)::*bla* PBAD*lolB* *araC* Δ*lolB* [pUA66::P*_cpxP_*-GFP] | LolB depletion, stress response reporter assay | This Study |
| KL819 | NR756 ∆(λ*att-lom*)::*bla* PBAD*lolB* *araC* Δ*lolB* [pUA66::P*_osmB_*-GFP] | LolB depletion, stress response reporter assay | This Study |
| KL820 | NR756 ∆(λ*att-lom*)::*bla* PBAD*lolB* *araC* Δ*lolB* [pUA66::P*_micA_*-GFP] | LolB depletion, stress response reporter assay | This Study |
| KL821 | NR756 ∆(λ*att-lom*)::*bla* PBAD*lolB* *araC* Δ*lolB* [pUA66::P*_rpoD_*-GFP] | LolB depletion, stress response reporter assay | This Study |
| KL981 | NR756 ∆(λ*att-lom*)::*bla* PBAD*lolB araC* Δ*lolB* *nlpE::spec* [pUA66::P*_cpxP_*-GFP] | LolB depletion, NlpE deletion, stress response reporter assay | This Study |
|  |  |  |  |
| Antibioitic Treatment Stress GFP Reporter Strains | | | |
| KL754 | MG3178 Δ*tolC* [pUA66::P*_osmB_*-GFP] | Stress response reporter assay, treatment with inhibitors | This Study |
| KL756 | MG3178 Δ*tolC* [pUA66::P*_micA_*-GFP] | Stress response reporter assay, treatment with inhibitors | This Study |
| MG4301 | MG3178 Δ*tolC* [pUA66::P*_cpxP_*-GFP] | Stress response reporter assay, treatment with inhibitors | This Study |
| MG4307 | MG3178 Δ*tolC* [pUA66::P*_rpoD_*-GFP] | Stress response reporter assay, treatment with inhibitors | This Study |
|  |  |  |  |
| Antibiotic Checkerboard Strains | | | |
| KL641 | NR756 ∆*lspA::kan* [pBAD30::*lspA*] | Depletion checkerboard | This Study |
| KL324 | NR756 ∆(λ*att-lom*)::*bla* PBAD*lolB* *araC* Δ*lolB* | Depletion checkerboard | This Study |
| KL326 | NR756 ∆*lolCDE::cam* [pBAD18::*lolCDE*] | Depletion checkerboard | This Study |
| KL328 | NR756 ∆*lolA::kan* [pBAD30::*lolA*] | Depletion checkerboard | This Study |
| MG4169 | NR756 *tolC::Tn10* | Checkerboard | This Study |
|  |  |  |  |
| MTSES Checkerboard Strains | | | |
| HS166 | NR756 ∆*lolA::kan* [pBAD18::*lolA(V24C)*-strep] | LolA mutant, MTSES treatment assay | This Study |
| HS254 | NR756 ∆*lolA::kan* [pBAD18::*lolA*-strep] | WT LolA, MTSES treamtent assay | This Study |
| HS322 | NR756 Δ*lolA* [pBAD18::*lolA(V24C)*-strep] [pUA66::P*_cpxP_*] | MTSES GFP reporter assay | This Study |
| HS323 | NR756 Δ*lolA* [pBAD18::*lolA(V24C)*-strep] [pUA66::P*_osmB_*] | MTSES GFP reporter assay | This Study |
| HS324 | NR756 Δ*lolA* [pBAD18::*lolA(V24C)-*strep] [pUA66::P*_rpoD_*] | MTSES GFP reporter assay | This Study |
| HS325 | NR756 Δ*lolA* [pBAD18::*lolA*-strep] [pUA66::P*_cpxP_*] | MTSES GFP reporter assay | This Study |
| HS326 | NR756 Δ*lolA* [pBAD18::*lolA*-strep] [pUA66::P*_osmB_*] | MTSES GFP reporter assay | This Study |
| HS327 | NR756 Δ*lolA* [pBAD18::*lolA*-strep] [pUA66::P*_rpoD_*] | MTSES GFP reporter assay | This Study |
| HS336 | NR756 Δ*lolA* [pBAD18::*lolA(V24C)*-strep] [pUA66::P*_micA_*] | MTSES GFP reporter assay | This Study |
| HS337 | NR756 Δ*lolA* [pBAD18::*lolA*-strep] [pUA66::P*_micA_*] | MTSES GFP reporter assay | This Study |
|  |  |  |  |
| Viability Assays | | | |
| MG4264 | MG3178 ∆*lgt::kan* [pCHAP9231] | Viability assays | 4 |
| MG4220 | MG3178 ∆*lgt::kan* ∆*lpp* [pCHAP9231] | Viability assays | This Study |
| MG4262 | MG3178 ∆*lspA::kan* [pBAD30::*lspA*] | Viability assays | 5 |
| MG4376 | MG3178 ∆*lspA::kan* Δ*lpp* [pBAD30::*lspA*] (with MG4262) | Viability assays | This Study |
| MG4012 | MG3178 *gut::Kan-rrnB* TT-araC-PBAD-*lnt* ∆*lnt::spec* | Viability assays | 6 |
| MG4243 | MG3178 *gut::Kan-rrnB* TT-*araC*-PBAD-*lnt* ∆*lnt:spec* ∆*lpp* | Viability assays | This Study |
| MG4249 | MG3178 ∆*lolCDE::cam* [pBAD18::*lolCDE*] | Viability assays | 7 |
| MG4251 | MG3178 ∆*lolCDE::cam* ∆*lpp* [pBAD18::*lolCDE*] | Viability assays | This Study |
| MG3856 | MG3178 ∆(λ*att-lom*)::*bla* PBAD*lolA araC* ∆*lolA::kan* | Viability assays | This Study |
| MG3857 | MG3178 ∆(λ*att-lom*)::bla PBAD*lolA araC* ∆*lolA::kan* ∆*lpp* | Viability assays | This Study |
| MG2162 | MG3178 Δ*lolB* [pBAD18::*lolB*] | Viability assays | 7 |
| MG2164 | MG3178 Δ*lolB* Δ*lpp::kan* [pBAD18::*lolB*] | Viability assays | 7 |
|  |  |  |  |
| MreB Strains | | | |
| KL82 | MG3178 ∆*rcsB* ∆*lpp* *zii::Tn10* *cpxA*24 *kan-mreB*(WT)-*msf*GFP | WT MreB bypass strain, checkerboards | This Study |
| KL83 | MG3178 ∆*rcsB* ∆*lpp* *zii::Tn10* *cpxA*24 Δ*lolB* Δ*lolA* *kan-mreB(E143A)-msfGFP* | Resistant MreB bypass strain, checkerboards | This Study |
| 1. Casadaban, M. J. Transposition and fusion of the *lac* genes to selected promoters in *Escherichia coli* using bacteriophage lambda and Mu. *Journal of Molecular Biology*. 104, 541-555 (1976). | | | |
|  |  |  |  |
| 2. Button, J. E., Silhavy, T. J., Ruiz, N. A suppressor of cell death caused by the loss of sigmaE downregulates extracytoplasmic stress responses and outer membrane vesicle production in *Escherichia coli. Journal of Bacteriology* 189(5), 1523-1530 (2007). | | | |
|  |  |  |  |
| 3. May, K. L., Lehman, K. M., Mitchell, A. M. & Grabowicz, M. A stress response monitoring lipoprotein trafficking to the outer membrane. *mBio* 10, (2019). | | |  |
|  |  |  |  |
| 4. Pailler, J., Aucher, W., Pires, M. & Buddelmeijer, N. Phosphatidylglycerol::Prolipoprotein Diacylglyceryl Transferase (Lgt) of *Escherichia coli* has seven transmembrane segments, and its essential residues are embedded in the membrane. *Journal of Bacteriology* 194, 2142–2151 (2012). | | | |
|  |  |  |  |
| 5. Xiao, Y. & Wall, D. Genetic redundancy, proximity, and functionality of *lspA*, the target of antibiotic TA, in the *Myxococcus xanthus* producer strain. *Journal of Bacteriology* 196, 1174–1183 (2014). | | | |
|  |  |  |  |
| 6. Armbruster, K. M. & Meredith, T. C. Identification of the Lyso-Form *N*-Acyl Intramolecular Transferase in Low-GC Firmicutes. *Journal of Bacteriology* 199, (2017). | | | |
|  |  |  |  |
| 7. Grabowicz, M. & Silhavy, T. J. Redefining the essential trafficking pathway for outer membrane lipoproteins. *Proceedings of the National Academy of Sciences* 114, 4769–4774 (2017). | | | |
