# Supplementary material for: A Biological Signature for the Inhibition of Outer Membrane Lipoprotein Biogenesis
Source: mBio. 2022 Jun 13;13(3):e00757-22. doi: 10.1128/mbio.00757-22 (PMC9239194; doi:10.1128/mbio.00757-22)
Supplement: TABLE S2 [file mbio.00757-22-s0010.docx]

**Table S2: Plasmid and Oligonucleotide List**

| **Plasmids used in this study** | | |
| --- | --- | --- |
| **Plasmid** | **Use** | **Reference** |
| pUA66::P*_cpxP_*-GFP | Cpx-responsive GFP transcriptional reporter; Kan-R | This Study |
| pUA66::P*_rpoD_*-GFP | RpoD-responsive GFP transcriptional reporter; Kan-R | This Study |
| pUA66::P*_osmB_*-GFP | RcsCpx-responsive GFP transcriptional reporter; Kan-R | This Study |
| pUA66::P*_micA_*-GFP | RpoE-responsive GFP transcriptional reporter; Kan-R | 1 |
| pCHAP9231 | Arabinose-inducible production of Lgt; Cam-R | 2 |
| pBAD30::*lspA* | Arabinose-inducible production of E. coli LspA; Amp-R | 3 |
| pBAD18::*lolCDE* | Arabinose-inducible production of LolCDE; Amp-R | 4 |
| pBAD30::*lolA* | Arabinose-inducible production of LolA; Amp-R | This Study |
| pBAD18::*lolA*-strep | Arabinose-inducible production of Strep-tagged LolA; Amp-R | This Study |
| pBAD18::*lolA(V24C)*-strep | Arabinose-inducible production of Strep-tagged LolA(V24C); Amp-R | This Study |
| pBAD18::*lolB* | Arabinose-inducible production of LolB; Amp-R | 4 |
| pCP20 | Flipping out kanamycin markers from Keio collection strains; t.s. Amp-R Cam-R | 5 |

| **Oligonucleotides used in this study** | | | |
| --- | --- | --- | --- |
| **Name** | **Sequence (5'-3')** | | **Description** |
| pUArpoD_vectF | tggtacaaatGGATCCTCTAGATTTAAGAAG | Gibson assembly of pUA66::P*_rpoD_*-GFP | |
| pUArpoD_vectR | ctgcggaacaACTCGAGGTGAAGACGAAAG | Gibson assembly of pUA66::P*_rpoD_*-GFP | |
| PrpoD12_F | cacctcgagtTGTTCCGCAGCTAAAACG | Gibson assembly of pUA66::P*_rpoD_*-GFP | |
| PrpoD12_R | tagaggatccATTTGTACCACGATAGTGC | Gibson assembly of pUA66::P*_rpoD_*-GFP | |
| pUAcpxP_vecR | tgacttccctattACTCGAGGTGAAGACGAAAG | Gibson assembly of pUA66::P*_cpxP_*-GFP | |
| pUAcpxP_promF | ttcacctcgagtAATAGGGAAGTCAGCTCTC | Gibson assembly of pUA66::P*_cpxP_*-GFP | |
| pUAcpxP_promR | atctagaggatccAACGATAGAGAGTTTACGATTC | Gibson assembly of pUA66::P*_cpxP_*-GFP | |
| pUAcpxP_vecF | ctctctatcgttGGATCCTCTAGATTTAAGAAGG | Gibson assembly of pUA66::P*_cpxP_*-GFP | |
|  |  |  | |
| pUA_osmBvec_F | tattatcagcggatcctctagatttaagaag | Gibson assembly of pUA66::P*_osmB_*-GFP | |
| pUA_osmBvec_R | atgatttgccactcgaggtgaagacgaaag | Gibson assembly of pUA66::P*_osmB_*-GFP | |
| PosmB_F | cacctcgagtggcaaatcatccgctctaag | Gibson assembly of pUA66::P*_osmB_*-GFP | |
| PosmB_R | tagaggatccgctgataataattttatatcttgagagtg | Gibson assembly of pUA66::P*_osmB_*-GFP | |
| p18LolA_V24C_F | TGCACTGACGGTAGCGGC | V24C substitution | |
| p18LolA_V24C_R | TTTTTGTGTGAAGCTGGCGTGGAAGC | V24C substitution | |

| 1. Mutalik, V. K., Nonaka, G., Ades, S. E., Rhodius, V. A. & Gross, C. A. Promoter strength properties of the complete sigma E regulon of Escherichia coli and Salmonella enterica. Journal of Bacteriology 191, 7279–7287 (2009). |
| --- |
| 2. Pailler, J., Aucher, W., Pires, M. & Buddelmeijer, N. Phosphatidylglycerol::Prolipoprotein Diacylglyceryl Transferase (Lgt) of Escherichia coli Has Seven Transmembrane Segments, and Its Essential Residues Are Embedded in the Membrane. Journal of Bacteriology 194, 2142–2151 (2012). |
| 3. Xiao, Y. & Wall, D. Genetic redundancy, proximity, and functionality of lspA, the target of antibiotic TA, in the Myxococcus xanthus producer strain. Journal of Bacteriology 196, 1174–1183 (2014). |
| 4. Grabowicz, M. & Silhavy, T. J. Redefining the essential trafficking pathway for outer membrane lipoproteins. Proceedings of the National Academy of Sciences of the United States of America 114, 4769–4774 (2017). |
| 5. Datsenko, K. A. & Wanner, B. L. One-step inactivation of chromosomal genes in Escherichia coli K-12 using PCR products. Proceedings of the National Academy of Sciences 97, 6640–6645 (2000). |
